# DeepSeek is open-access and the next AI disrupter for radiology

**DOI:** 10.1093/radadv/umaf009

**Published:** 2025-02-18

**Authors:** Yifan Peng, Qingyu Chen, George Shih

**Affiliations:** 1 Department of Population Health Sciences, Weill Cornell Medicine, New York, NY 10022, United States; 2 Department of Biomedical Informatics and Data Science, Yale School of Medicine, New Haven, CT 06510, United States; 3 Department of Radiology, Weill Cornell Medicine, New York, NY 10065, United States

**Keywords:** DeepSeek, open-weight, radiology

## Introduction

Recent years have seen the rapid integration of artificial intelligence (AI) into the field of radiology. The most recent potential one is DeepSeek, which is redefining the benchmarks for open-weight, computational efficiency, and problem-solving capabilities.^1^ The emergence of DeepSeek has ignited enthusiasm as well as consternation, causing stock market volatility with its economic implications.^2^ More importantly, its technical capabilities have generated extensive discussions in the engineering and biomedical domains.^3^ Here, we briefly discuss what sets DeepSeek apart and why it could be the next tool in the AI transformation of radiology.

## Innovations brought by DeepSeek

DeepSeek is an AI startup based in Hangzhou, China. It is known for releasing various models, including DeepSeek-V3 and DeepSeek-R1. DeepSeek-V3 is targeted to compete directly with OpenAI’s GPT-4o, whereas DeepSeek-R1 is positioned against OpenAI's o1, as shown in Table 1.

One notable distinction of DeepSeek models is their open nature, which includes the models and weights, although not the training data and training code.^1,4^ It allows everyone to examine their training processes in detail. From a technological standpoint, DeepSeek-R1 employed the multistage training approach. It began with a “cold start” phase, focusing on fine-tuning with a small set of carefully crafted examples to enhance clarity and readability. Subsequently, the models underwent additional reinforcement learning and refinement steps, including rejection of low-quality outputs based on human preference and verifiable rewards. This resulted in models that reason effectively while delivering polished and consistent answers.

To reduce the training cost (only $5.576 M^1^) the DeepSeek team implemented FP8 training^4^ and Mixture of Experts. FP8 is a progression from 16-bit data formats.^5^ Here, DeepSeek models used a mixed precision framework for training, where most compute-density operations are conducted in FP8. In contrast, a select few key operations are strategically retained in their original data formats to balance training efficiency and numerical stability. The Mixture of Experts technique utilizes several expert networks to divide the problem space into homogeneous regions, optimizing problem-solving. Consequently, DeepSeek requires less computing power than previous models. It has been reported that a single server equipped with eight H200 GPUs can effectively run the full version of DeepSeek-R1.^6^

In addition, DeepSeek introduces Multi-head Latent Attention, which reduces overhead by transforming the key-value cache required by standard Multi-head Attention into a latent vector. This approach significantly improves inference efficiency and scales well across small and large Mixture of Experts models.

## How could radiology benefit from DeepSeek?

One notable feature of DeepSeek-R1 is “chain-of-thought” reasoning (CoT). This technique guides large language models (LLMs) to follow a reasoning process when dealing with complex problems. Although CoT can be applied with other LLMs, they often require users to carefully construct CoT prompts (eg, explain the reasoning), posing a barrier to lay end users.

DeepSeek uses large-scale reinforcement learning, reward modeling, and distillation to enhance reasoning performance. When we chatted with DeepSeek-R1, we did not immediately get a response. The model first uses CoT reasoning to consider the problem. Only once it finishes thinking does it start outputting the answer. We also observed that DeepSeek-R1 is capable of self-reflection, criticism, and correction. This has potential for radiology applications, where synthesizing different data modalities and effective reasoning are essential.

To assess the capabilities of DeepSeek and other LLMs, we challenged them with a multiple-choice disease classification problem designed to identify specific abnormal findings from radiology reports. In this setup, the input is a radiology report and a list of candidate answers representing potential diagnoses (detailed in Supplementary Materials). Our case examples were crafted using synthetic data created by radiologists, ensuring that no patient information was used.

These samples demonstrate that DeepSeek-R1 outperforms Llama-3.3–70b; however, we did not find significant advantages over GPT-4o. The open-weight nature of DeepSeek-R1 and its implementation locally suggest that developing reasonably sized LLM models is feasible without sacrificing high functionality. This observation is expected and may reflect similar performance in other tasks.

It is important to note that our evaluation encompassed only a limited number of examples. A more extensive comparison across a broader range of tasks could yield more profound insights into DeepSeek's true capabilities.

## Future challenges

Using DeepSeek to analyze radiology reports presents several challenges and ethical considerations. As with all deep learning platforms, data privacy is a primary concern. Currently, several governments have banned DeepSeek from government devices (e.g., New York State,^7^ Texas^8^) because of perceived security risks. However, unlike the other LLMs, DeepSeek’s open-weight nature and low resource requirements make it feasible for enterprises like large health care organizations to run the model locally for in-house AI training and implementation. Consequently, DeepSeek-V3 and DeepSeek-R1 are expected to foster collaborative environments and accelerate AI innovation. However, the release of DeepSeek-R1 raises important questions, particularly regarding the curation of datasets^9^ and the absence of training code, which are critical for transparency and responsible use.

In addition, while DeepSeek emphasizes reasoning, we have observed that its responses tend to be excessively verbose, with many details that can quickly overwhelm users tasked with reading, reviewing, or approving them. This raises an important question: Is the detailed reasoning in responses truly beneficial, and to what extent does it add value? This remains an open area of inquiry.

Third, LLMs, including DeepSeek, still require comprehensive evaluation across several dimensions, such as Quality of Information, Understanding and Reasoning, Expression Style and Persona, Safety and Harm, and Trust and Confidence.^10^ To date, no publicly reported studies specifically explore DeepSeek's capabilities in analyzing clinical text. This underscores the need for further investigation to understand and validate its potential in these areas fully.

## Conclusion

DeepSeek has disrupted both the AI industry and academia and is expected to act as a catalyst for further development in the field. These advanced capabilities will definitely promote the successful integration of AI in radiology in the near future.

As DeepSeek and other LLMs, both proprietary and open, gain prominence, there is an increasing demand for a collaborative co-design approach to integrating LLMs into clinical domains. This approach should encompass diverse stakeholders, including technology developers, ethicists, radiology domain experts, and end-users, to ensure these models effectively address real-world human needs and reflect societal values.

## Supplementary Material

Supplementary Materials

Supplementary material

Supplementary material is available at *Radiology Advances* online.

## Figures and Tables

**Table 1.** Comparisons between GPT-4o, Open AI o1, Llama 3.3, DeepSeek-v3, and DeepSeek-R1.

	GPT-4o	OpenAI o1	Llama 3.3	DeepSeek-V3	DeepSeek-R1

**Date of Release**	May 13, 2024	December 5, 2024	December 6, 2024	December 26, 2024	January 20, 2025
**Design**					
**Number of parameters**	Not disclosed	Not disclosed	70B	671B	671B
**Input modality**	Text, Image, Audio	Text, Image	Text	Text	Text
**Training dData**	Not disclosed	Not disclosed	A new mix of publicly available online data.	Not disclosed	Not disclosed
**Training strategy**	Not disclosed	Not disclosed	SFT, RL	SFT, RL, FP8	SFT, RL, Multistage training
**Context window**	128k	200k	128k	128k	128k
**GPU hours for training**	Not disclosed	Not disclosed	7.0M	2.79M	Not disclosed
**Evaluation**					
**MMLU**	88.7	92.3	86	88.5	90.8
**Cost**					
**Input**	$1.25/1M cached	$7.50/1M cached	Free	Free; API: $0.014/1M cached	Free; API: $0.14/1M cached
**Output**	$10.00/1M	$60.00/1M	Free	Free; API: $0.28/1M	Free; API: $2.19/1M
**Usage**					
**Availability**	Private (OpenAI)	Private (OpenAI)	Open	Open	Open
**Run locally?**	API	API	Yes	Yes	Yes
**License**	Proprietary	Proprietary	Meta Llama Community License Agreement	MIT license	MIT license

Abbreviations: API = application programming interface, MMLU = massive multitask language understanding, SFT = supervised fine-tuning, RL = reinforcement learning, FP8 = 8-bit floating point.
