# Supplementary material for: DeepSeek is open-access and the next AI disrupter for radiology
Source: Radiol Adv. 2025 Feb 18;2(1):umaf009. doi: 10.1093/radadv/umaf009 (PMC12363671; doi:10.1093/radadv/umaf009)
Supplement: umaf009_Supplementary_Data [file umaf009_Supplementary_Data.zip › Supplementary_materials.pdf]

DeepSeek is open-access and the next AI disrupter for radiology
Supplementary materials

Prompt:

You are given a clinical report. Your task is to identify any diseases mentioned in the report and list them. The result list should be formatted exactly as [disease1, disease2, ...] and be on a single line.

Only use diseases from this list, there are 44 diseases. If there are no diseases, output 'Normal'
['Support Devices', 'Adenopathy', 'Atelectasis', 'Azygos Lobe', 'Bulla', 'Calcification of the Aorta', 'Cardiomegaly', 'Cardiomyopathy', 'Clavicle Fracture', 'Consolidation',
'Edema', 'Emphysema', 'Enlarged Cardiomediastinum', 'Fibrosis', 'Fissure', 'Fracture', 'Granuloma', 'Hernia', 'Hilum', 'Hydropneumothorax', 'Infarction',
'Infiltration', 'Kyphosis', 'Lobar Atelectasis', 'Lung Lesion', 'Lung Opacity', 'Mass', 'Nodule', 'Osteopenia', 'Pleural Effusion', 'Pleural Other',
'Pleural Thickening', 'Pneumomediastinum', 'Pneumonia', 'Pneumoperitoneum', 'Pneumothorax', 'Pulmonary Embolism', 'Pulmonary Hypertension', 'Rib Fracture',
'Round(ed) Atelectasis', 'Scoliosis', 'Subcutaneous Emphysema', 'Tortuous Aorta', 'Tuberculosis']

Guidelines:
* Be careful about negation. E.g., do not include Pneumonia if the text says "no Pneumonia"
* Only include diseases that are certain. Entities that are 'likely', 'possibly' should not be returned.
* Provide the list first. Followed by reasoning starting next line

Example Reports and Outputs:

1. Report: 'Lung volumes are markedly low. This results in exaggeration of the cardiac\n silhouette size which is borderline enlarged. Mediastinal and hilar contours\n are unremarkable. Crowding of the bronchovascular structures is present\n without overt pulmonary edema. Patchy and linear opacities in the lung bases\n most likely are reflective of atelectasis. No focal consolidation, pleural\n effusion or pneumothorax is identified. No acute osseous abnormality is\n visualized.'
Output:
['Lung Opacity']
Reason: The report mentions 'Patchy and linear opacities in the lung bases'

2. Report: 'The lungs are clear without consolidation or edema. There is no\n pleural effusion or pneumothorax. There is mild cardiomegaly. The\n mediastinal contours are normal. The vertebral body heights are maintained in\n the thoracic spine. No rib fractures identified.'
Output:
['Cardiomegaly']
Reason: The report mentions 'There is mild cardiomegaly'

3. Report: 'The lungs are clear without focal consolidation. No pleural effusion or\n pneumothorax is seen. The cardiac and mediastinal silhouettes are stable.\n Hilar contours are stable. There is persistent elevation of the right\n hemidiaphragm with minor right basilar atelectasis.'
Output:
['Atelectasis']
Reason: The report mentions 'with minor right basilar atelectasis'

Report to Analyze:
{note}

Output:

Sample results

**Reports**	**DeepSeek-V3**	**DeepSeek-R1**	**GPT-4o**	**Llama-3 70b**
Exam: CXR<br>History: Shortness of breath<br>Comparison: 1 week prior<br>Findings: Swan Ganz catheter is present. Bilateral pulmonary edema is again seen with areas of bibasilar atelectasis. Enlarged cardiac silhouette is again seen.<br>Impression: Stable appearance of bilateral pulmonary edema and bibasilar atelectasis.	Atelectasis<br>Cardiomegaly<br>Edema	Atelectasis<br>Cardiomegaly<br>Edema<br>Support Devices	Atelectasis<br>Cardiomegaly<br>Edema<br>Support Devices	Atelectasis<br>Cardiomegaly<br>Pulmonary Edema
Exam: CXR<br>History: Pneumothorax, follow up<br>Comparison: 1 day ago	Pleural Effusion<br>Pneumothorax	Pleural Effusion<br>Pneumothorax<br>Support Devices	Pleural Effusion<br>Pneumothorax	Pleural Effusion<br>Pneumothorax

Findings: Two left apically directed chest tubes are not significantly changed in position. Left sided central venous catheter with tip projecting over the superior cavoatrial junction.<br>Decreased small to moderate left apical pneumothorax. Unchanged small left pleural effusion. No focal consolidation.<br>Redemonstrated sclerotic lobulated osseous lesion over the mid-clavicle, possibly myositis ossificans.<br>Impression: Decreased small to moderate left apical pneumothorax. Unchanged small left pleural effusion.				
Exam: CXR<br>History: Three days of fever, shortness of breath<br>Comparison: none<br>Findings: Bilateral peribronchial cuffing. Faint perihilar reticular opacities bilaterally. No pleural effusion or pneumothorax. The cardiomediastinal silhouette is normal.<br>Cholecystectomy.<br>Impression: Peribronchial cuffing with faint perihilar reticular opacities bilaterally, probably representing viral pneumonia/small airways disease.	Normal	Lung Opacity	Lung Opacity	Pneumonia
Exam: CXR (AP)<br>History: evaluate NG tube position, fever, cough<br>Comparison: 3 days prior<br>Findings: Interval placement of NG tube which terminates in the stomach. As seen on prior, there is a right PICC terminating at the cavoatrial junction and left IJ terminating in the SVC. There is focal consolidation of the left mid lung field and bilateral patchy opacities, which appear slightly increased since previous exam. No signs of pneumothorax. Cardiomediastinal silhouette is stable.<br>Impression: Normal position of NG tube. Worsening left	Consolidation<br>Lung Opacity	Consolidation<br>Support Devices	Consolidation<br>Lung Opacity	Consolidation<br>Lung Opacity

mid lung focal consolidation and bilateral patchy opacities which may represent ongoing infection.				
Exam: CXR (PA)<br>History: persistent SOB, prior pneumothorax<br>Comparison: 1 week prior<br>Findings: Interval placement of right side chest tube. Interval resolution of right pneumothorax. There is a persistent moderate left pleural effusion, improved. No focal consolidation or signs of pulmonary edema. Cardiomediastinal silhouette is stable.<br>Impression: Resolution of right-sided pneumothorax. Improving left sided pleural effusion.	Pleural Effusion	Pleural Effusion<br>Support Devices	Pleural Effusion<br>Pneumothorax	Pleural Effusion<br>Pneumothorax
